# Examining the BMI-mortality relationship using fractional polynomials

**DOI:** 10.1186/1471-2288-11-175

**Published:** 2011-12-28

**Authors:** Edwin S Wong, Bruce CM Wang, Louis P Garrison, Rafael Alfonso-Cristancho, David R Flum, David E Arterburn, Sean D Sullivan

**Affiliations:** 1 Pharmaceutical Outcomes Research and Policy Program, University of Washington, Seattle, WA, USA; 2 Northwest Center for Outcomes Research in Older Adults, VA Puget Sound Health Care System, Seattle, WA, USA; 3 Surgical Outcomes Research Center, University of Washington, Seattle, WA USA; 4 Group Health Research Institute, Seattle, WA

## Abstract

**Background:**

Many previous studies estimating the relationship between body mass index (BMI) and mortality impose assumptions regarding the functional form for BMI and result in conflicting findings. This study investigated a flexible data driven modelling approach to determine the nonlinear and asymmetric functional form for BMI used to examine the relationship between mortality and obesity. This approach was then compared against other commonly used regression models.

**Methods:**

This study used data from the National Health Interview Survey, between 1997 and 2000. Respondents were linked to the National Death Index with mortality follow-up through 2005. We estimated 5-year all-cause mortality for adults over age 18 using the logistic regression model adjusting for BMI, age and smoking status. All analyses were stratified by sex. The multivariable fractional polynomials (MFP) procedure was employed to determine the best fitting functional form for BMI and evaluated against the model that includes linear and quadratic terms for BMI and the model that groups BMI into standard weight status categories using a deviance difference test. Estimated BMI-mortality curves across models were then compared graphically.

**Results:**

The best fitting adjustment model contained the powers -1 and -2 for BMI. The relationship between 5-year mortality and BMI when estimated using the MFP approach exhibited a J-shaped pattern for women and a U-shaped pattern for men. A deviance difference test showed a statistically significant improvement in model fit compared to other BMI functions. We found important differences between the MFP model and other commonly used models with regard to the shape and nadir of the BMI-mortality curve and mortality estimates.

**Conclusions:**

The MFP approach provides a robust alternative to categorization or conventional linear-quadratic models for BMI, which limit the number of curve shapes. The approach is potentially useful in estimating the relationship between the full spectrum of BMI values and other health outcomes, or costs.

## Background

Obesity and its impact on health and the healthcare system is one of the most important public health issues Western society faces. Many studies have measured the detrimental effect of obesity on life expectancy by estimating the relationship between mortality and body mass index (BMI) [weight (kg)/height^2 ^(m^2^)]. However, the evidence is mixed as to the exact relationship. While some studies have concluded no relation [1], an inverse relation [2] or a direct relation [3], the majority of studies have identified a U-shaped relation [4-10] or a J-shaped relation [11-15]. One reason for these differences is the wide array of datasets used in the analyses. However, the results in these studies are also sensitive to assumptions regarding the estimation sample and functional form for BMI.

The vast majority of studies have employed a non-parametric approach, by treating BMI as a categorical variable. The mortality risk of individuals in different BMI groups is computed relative to a reference BMI category. World Health Organization (WHO) BMI classifications [16] are typically used. For example, numerous studies have computed the excess mortality due to being overweight (BMI = 25-30) and obese (BMI ≥ 30) [17-22]. Categorizing continuous variables has been a popular approach, particularly because of *a priori *knowledge that the relationship between the two measures is nonlinear. However, Royston, et al. (2006) [23] pointed out a number of disadvantages with this approach. The most significant drawback is the loss of information and power through what is equivalent to rounding. For example, studies of excess mortality using WHO BMI classification implicitly assume all individuals that are in the normal category (BMI = 18.5-25) exhibit the same mortality risk. However, the normal category is heterogenous and includes individuals who are healthy and those who are chronically ill. Categorization is particularly problematic if groups are large. In addition, the number of cutoff points and where to place cutoff points is arbitrary. Finally, results are not necessarily robust across the choice of reference category.

A number of studies have used other approaches that maintain BMI as a continuous variable. Estimation of the BMI-mortality curve using a continuous measure is problematic because the relationship is nonlinear and the distribution of BMI is right skewed. Schauer et al. (2010) [24] included linear and squared terms to account for nonlinearities, but truncated their sample to respondents with a BMI of 25 or greater to address the skewness in BMI. Durazo et al. (1998) [6] transformed BMI into a normally distributed variable using Tukey's "ladder of powers" method. Gronniger et al. (2006) [25] treated BMI non-parametrically without categorization.

The purpose of this study was to investigate a flexible approach to modelling the nonlinear and asymmetric relationship between adult mortality and obesity measured using BMI. We implemented the multivariable fractional polynomials (MFP) method [26,27] and maintained BMI as a continuous variable. Instead of imposing a specific functional form, the MFP method allows the data to determine the best fitting functional form for BMI and other adjustment variables. We hypothesized that this method would provide the ability to capture the relationship between mortality and BMI in a compact, parsimonious model. The MFP performance and results were then compared against other commonly used regression models that estimate the BMI-mortality relationship.

## Methods

The data used in this study were from the NHIS, publicly available through the Centers for Disease Control and Prevention (CDC). The NHIS is a nationally representative cross-sectional household survey covering the non-institutionalized civilian population in the United States (U.S.) and is conducted annually [28]. Households and non-institutional sample units with special living arrangements (e.g. dormitories, boarding houses) were randomly sampled. For each unit sampled, a randomly selected adult and child (if present) were used to collect core health information. Beginning in 1997, individuals aged 65 and above who were black, Hispanic or Asian were oversampled. We combined data from 1997 through 2000. Our sample was linked to the National Death Index (NDI) with mortality follow-up through December 31, 2005 ensuring all respondents were tracked for at least five years after completing the NHIS. Self-reported weight and height measurements without shoes were used to construct BMI. Smoking history was a dichotomous variable indicating whether an individual has ever smoked. Individuals below the age of 18 and under 18.5 BMI were excluded from our analysis. We also excluded 4,599 respondents who have missing BMI measurements or have a BMI of over 99.99, whose observations were truncated, and 229 observations with unknown smoking status. The final sample contained 117,961 respondents.

We investigated the relationship between mortality and obesity through BMI using the logistic regression model, stratified by gender and adjusting for age and smoking history. The logistic model was chosen over the Cox proportional hazards model because the proportional hazards condition did not hold for the BMI fractional polynomial (FP) terms. We used 5-year all-cause mortality as the dependent outcome because of the very low incidence of death annually. To check for robustness, we also estimated all models using 3-year mortality as the outcome, which produced similar results. Analyses were stratified by gender because the biological process by which men and women gain and maintain weight is different [29]. We adjusted for smoking status because it confounded the BMI-mortality relationship, which if ignored may result in overestimation of the BMI associated with minimum mortality [30]. Sample adult weights from the NHIS, which denoted the inverse probability of inclusion into the sample were used within the logistic regression model to correct for potential biases resulting from the NHIS sampling design. Because data were pooled, sampling weights were divided by the number of years to generate a sample that is representative of the U.S. population on average from 1997-2000.

We maintained BMI as a continuous variable in our analysis. To account for the nonlinear and asymmetric relationship between BMI and mortality, we first applied the fractional polynomials [31,32] method. To allow for flexibility in fitting a curve with a single turning point, we considered second degree polynomial transformations for BMI. We used the closed test procedure [33] which first determined the best fitting second degree polynomial by choosing power transformations from the set {-2, -1, -0.5, 0, 0.5, 1, 2, 3}, where 0 denotes the log transformation. The best fitting second-degree FP was then compared against the null model using a deviance difference test with four degrees of freedom to determine whether BMI should be included in the model. If the first test was statistically significant, a second deviance difference test with three degrees of freedom was applied to compare the best fitting second degree FP against the linear model. If the second test was significant, a final deviance difference test with two degrees of freedom compared the best fitting second degree FP with the best fitting first degree FP. If the final test was significant, the second degree FP was included, otherwise the first degree FP was chosen. To prevent collinearity and model overfit, the best fitting first degree polynomial was chosen for age. The selection of powers for BMI and age was computed simultaneously using the multivariable fractional polynomials (MFP) method [26,27], which combined backward elimination to select the best fitting model. The regression model we estimated was

$$\begin{array}{c} \mathrm{logit}\left(\pi_{i}\right)=\;\beta_{0}+\beta_{1}BMI^{p1}+\beta_{2}BMI^{p2}+\;\\ \;\beta_{3}AGE^{q1}+\beta_{4}SMOKE\; \end{array}$$

where *π_i _*was the 5-year death probability for individual *i*, *p1 *and *p2 *were the fractional powers for BMI, and *q1 *was the fractional power for age. The MFP method also scaled and centered variables in model selection process to improve numerical stability and to provide a model intercept that was easier to interpret. A nominal p-value of 0.05 was used to test all hypotheses. To evaluate the validity of the FP model for BMI, we graphically compared three models with the main FP model defined by (1). First, we estimated the model which categorized BMI into 30 narrow bins (1 bin for each BMI unit between 18 and 40, for every two BMI units between 40 and 54 and a single bin for BMI above 54) while also adjusting for age and smoking status. We then estimated separate FP models after omitting subjects with early death (< 1 year from baseline) and extreme BMI values (> 50).

Interactions between adjustment variables were tested to address the possibility of differences in the BMI-mortality curve across the age distribution, and by smoking history. The multivariable fractional polynomial interaction (MFPI) algorithm [26] was used to assess interactions, which first determined the best fitting polynomial functions for BMI and age using MFP and then tested for significant interactions between fractionally transformed variables and smoking history using a deviance difference test. We then verified interactions found by the MFPI algorithm graphically using Lowess smoothed curves. The use of FPs when fitting models using BMI as a continuous variable avoided inclusion of spurious interactions in a strictly linear model.

The BMI associated with minimum mortality was calculated by first estimating the final MFP model (including interaction terms) using logistic regression. To derive the optimal BMI, we set the first derivative of the estimated FP model equal to 0 and solved for BMI. As an example, the optimal BMI for the model with linear and quadratic BMI terms without interactions is $-{\hat{\beta }}_{1}∕\left(2{\hat{\beta }}_{2}\right)$, where ${\hat{\beta }}_{1}$ and ${\hat{\beta }}_{2}$ are the logistic regression coefficients for the linear and quadratic BMI terms, respectively. Confidence intervals were based on standard errors computed using the delta method.

We compared the BMI-mortality curves derived using the MFP method with the continuous BMI model containing linear and quadratic BMI terms and the categorical model based on WHO BMI classifications. Models were compared on the basis of model fit, the shape of the BMI-mortality curve, the magnitude and uncertainty in the BMI associated with minimum mortality and mortality estimates. All statistical models were fit using the STATA Statistical Software (Version 11; College Station, TX). The STATA procedure MFP was used to determine the functional forms for age and BMI and the procedure NLCOM was used to calculate estimates and confidence intervals for the BMI associated with minimum mortality.

## Results

Descriptive statistics for the NHIS sample used in this study are presented in Table 1. Our sample included 52,549 men and 65,412 women with a mean age of 45.49 and 47.13 years, respectively. The percentage of respondents who died within five years was 6.37% among men and 5.55% among women. The number of deaths per thousand individuals were similar across annual survey cohorts for both the male and female samples. The majority of the sample was in the normal and overweight BMI ranges. Ever smokers made up 54.93% of the male sample and 40.47% of the female sample.

**Table 1:** Descriptive statistics for NHIS adult cohort from 1997 to 2000 used to estimate the BMI-mortality relation

Survey Year	1997	1998	1999	2000	Total
**Male**					

Sample Size	14460	13103	12147	12839	52549

# Deaths (5-year)	984	914	835	837	3570

Deaths/1000 persons	68.05	69.76	68.74	65.19	67.94

Age (Mean)	45.17	45.58	45.83	45.44	45.49

BMI Level (% Prevalence)					

Normal: [18.5, 25)	37.14%	35.80%	35.11%	34.38%	35.66%

Overweight: [25, 30)	43.78%	43.88%	43.49%	44.31%	43.87%

Obese I: [30, 35)	14.10%	15.09%	15.49%	15.34%	14.97%

Obese II: [35, 40)	3.49%	3.83%	4.16%	4.28%	3.92%

Obese III: 40+	1.49%	1.40%	1.75%	1.70%	1.58%

Ever Smoker	56.20%	55.39%	54.91%	53.06%	54.93%

**Female**					

Sample Size	18210	15928	15340	15934	65412

# Deaths (5-year)	1002	926	845	858	3631

Deaths/1000 persons	55.02	58.14	55.08	53.85	55.51

Age (Mean)	46.80	47.36	47.35	47.08	47.13

BMI Level (% Prevalence)					

Normal: [18.5, 25)	49.75%	48.17%	47.37%	46.69%	48.06%

Overweight: [25, 30)	28.90%	29.36%	29.69%	29.32%	29.30%

Obese I: [30, 35)	13.45%	14.04%	14.49%	14.68%	14.14%

Obese II: [35, 40)	4.87%	5.20%	5.11%	5.62%	5.19%

Obese III: 40+	3.03%	3.22%	3.34%	3.69%	3.31%

Ever Smoker	40.96%	40.98%	40.23%	39.63%	40.47%

### Model Fit

**Figure 1:**
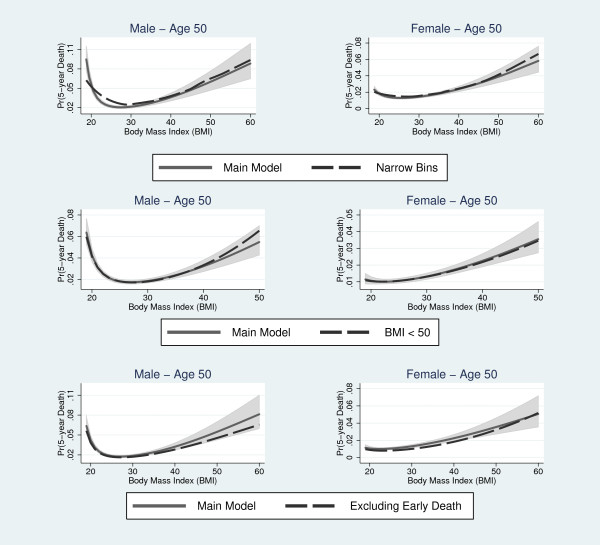
**Comparison of the main fractional polynomial model with BMI categorized into narrow bins (top row), the fractional polynomial model excluding extreme BMI values (middle row) and the fractional polynomial model excluding early deaths (bottom row)**. Shaded regions denote 95% confidence interval for the fractional polynomial model.

The best fitting model for BMI identified by the MFP procedure included the terms ${\bar{BMI}}^{-2}$ and ${\bar{BMI}}^{-2}*\text{ln}\left(\bar{BMI}\right)$ for the male sample and the terms ${\bar{BMI}}^{-2}$ and ${\bar{BMI}}^{-1}$ for the female sample, where $\bar{BMI}=BMI∕10$. In both samples, the best fitting model included a squared term for age. The main adjustment model contained smoking status, two BMI and one age polynomial terms. For men, the transformed model significantly improved model fit relative to the untransformed model (Deviance Difference = 231.79, *p-value *< 0.001), the linear-quadratic model (Deviance Difference = 105.62, *p-value *< 0.001) and the categorical model (Deviance Difference = 81.63, *p-value *< 0.001). Similar improvements in model fit were found in the female sample for the FP model relative to the untransformed model (Deviance Difference = 173.04, *p-value *< 0.001), the linear-quadratic model (Deviance Difference = 131.93, *p-value *< 0.001) and the categorical model (Deviance Difference = 82.11, *p-value *< 0.001). The FP model for BMI produced a similar BMI-mortality curve compared to the model categorizing BMI into narrow bins for both genders. Also, the BMI-mortality curves produced by the main FP model and the models omitting early deaths and extreme BMI values were similar in shape (Figure 1).

After finding the best fit for the main model, the age-smoking history (Deviance Difference = 15.88, *p-value *< 0.001) and BMI-age interactions (Deviance Difference = 35.31, *p-value *< 0.001) were both identified as statistically significant in the female sample. The final model was selected using forward selection. After including the age-BMI interactions, the age-smoking history interaction remained significant (Deviance Difference = 15.44, *p-value *< 0.001). Logistic regression results for the FP model, including significant interactions are in Table 2. We did not find statistically significant interactions in the male sample.

**Table 2:** Logistic regression coefficient estimates and standard errors in parentheses for the final adjustment model including smoking status, fractionally transformed BMI^1 ^and age^2 ^and interactions identified as significant

	Male	Female
BMI(p1)	24.260	20.328

	(1.938)	(5.186)

BMI(p2)	-49.284	-18.490

	(4.307)	(3.766)

Age(q1)	0.077	

	(0.001)	

Age(q1)*BMI(p1)		0.244

		(0.165)

Age(q1)*BMI(p2)		-0.115

		(0.125)

Age(q1) | Ever Smoked = 0		0.082

		(0.002)

Age(q1) | Ever Smoked = 1		0.075

		(0.002)

Ever Smoked	0.581	0.832

	(0.050)	(0.084)

Constant	-4.366	-4.712

	(0.054)	(0.075)

		

Log Likelihood	-9503.563	-10179.990

^1 ^For the male sample, $BMI\left(p1\right)={\left(\frac{BMI}{10}\right)}^{-2}$- 0.137, $BMI\left(p2\right)={\left(\frac{BMI}{10}\right)}^{-2}*\text{ln}\left(\frac{BMI}{10}\right)$ - 0.376 and for the female sample, $BMI\left(p1\right)={\left(\frac{BMI}{10}\right)}^{-2}$- 0.142, $BMI\left(p2\right)={\left(\frac{BMI}{10}\right)}^{-1}$- 0.376.

^2 ^For the male sample, $\text{Age}\left(q1\right)={\left(\frac{\text{Age}}{10}\right)}^{2}$- 20.692 and for the female sample, $\text{Age}\left(q1\right)={\left(\frac{\text{Age}}{10}\right)}^{2}$- 22.216.

**Figure 2:**
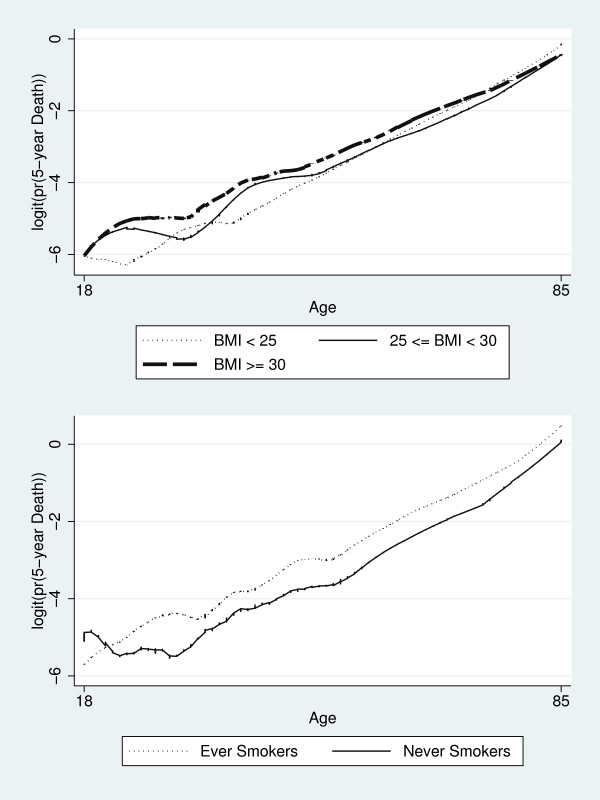
**Smoothed Lowess regression lines illustrating the BMI-age (top panel) and age-smoking status (bottom panel) interactions in the female sample**.

Overfit of the model may result in spurious interactions. We assessed interactions identified as significant graphically by performing Lowess smoothing on 5-year of death transformed into logits. Figure 2 shows the differential effect of age across smoking status and BMI, respectively, in the female sample. Differences in the slope of running smoothed lines across the age distribution confirmed the interactions identified as significant.

### BMI-Mortality Curve

**Figure 3:**
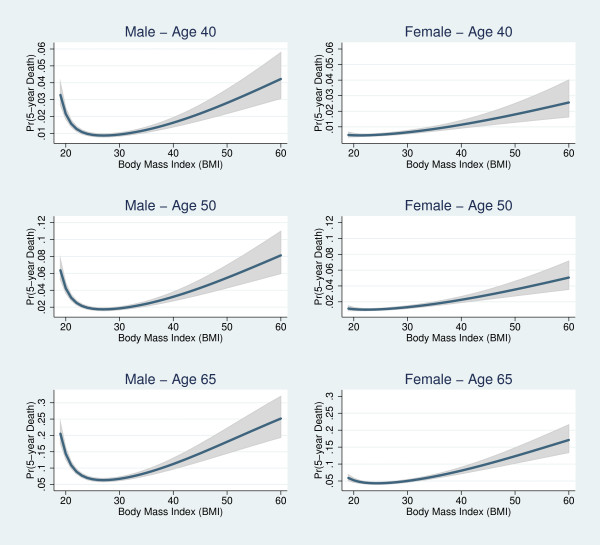
**Predicted mortality and 95% confidence interval based on the best fitting fractional polynomial model for male and female never smokers, age 40, 50 and 65**.

**Figure 4:**
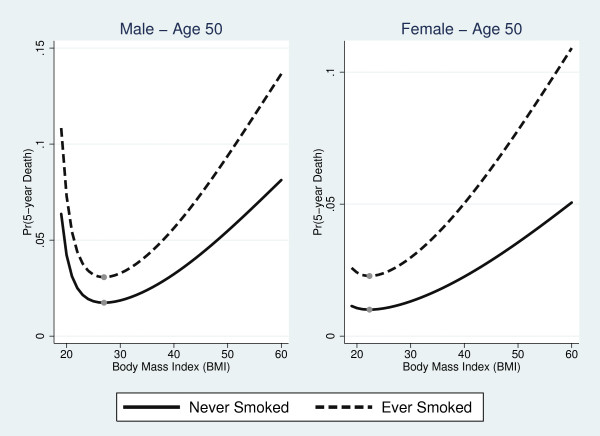
**Predicted mortality based on the best fitting fractional polynomial model for male and female ever smokers, age 40, 50 and 65**.

Fitted curves showing the relationship between the 5-year probability of death and the associated 95% confidence interval as a function of BMI are in Figure 3. We present curves for male and female non-smokers at ages 40, 50 and 65, respectively. For BMI 18.5 and above, the estimated relationship between BMI and mortality was J-shaped for women, but U-shaped for men. At all ages, the female curve was lower and less steep at both tails of the BMI distribution. Increases in age were associated with increases in mortality, however, fitted curves have the same shape across the age distribution. For both genders, the 5-year probability of death increased exponentially with BMI. Death probabilities increased rapidly starting at BMI = 40. The wide confidence intervals at the right tail of the BMI distribution stemmed from the low proportion of extremely obese (BMI ≥ 40) individuals in the population. Similar curve shapes were found for those who have ever smoked (Figure 4).

**Figure 5:**
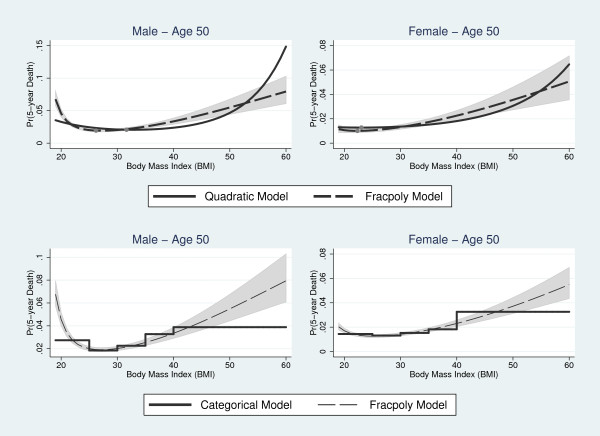
**Comparison of the best fitting fractional polynomial model with the linear-quadratic model for BMI (top row) and the categorical model (bottom row) for never smokers at age 50**. Shaded regions denote 95% confidence interval for the fractional polynomial model.

The top panel of Figure 5 compares the MFP and linear-quadratic BMI models for male and female never smokers, respectively, at age 50. For both genders, the BMI-mortality curve produced by the linear-quadratic model was J-shaped. The linear-quadratic overestimated mortality at the right tail of the BMI distribution and underestimated mortality in the 31-50 range for men and 30-52 BMI range for women when compared to the MFP model. In the male sample, the MFP model produced higher mortality estimates for subjects at the low end of the normal category.

The bottom panel of Figure 5 compares the MFP and categorical models for age 50 never smokers. The categorical approach matched MFP estimates closely in the overweight category for men. There was a lower degree of correspondence in BMI-mortality curves for all other BMI classifications. The categorical model also underestimated mortality at both tails of the BMI distribution.

### BMI Associated with Minimum Mortality

For the MFP model, the BMI associated with minimum mortality was 26.97 (95% Confidence Interval [CI], 26.41 to 27.54) (Figure 5) in the male sample. Because we did not identify significant interactions with BMI, the optimal BMI was constant for both smokers and non-smokers and across all ages. In contrast, the BMI associated with minimum mortality for women increased with age. At age 50, the optimal BMI was 22.34 (95% CI, 20.10 to 24.57). The optimal BMI ranged from 19.25 (95% CI, 13.31 to 25.18) at age 18 to 26.86 (95% CI, 25.70 to 28.02) at age 85. The BMI associated with minimum mortality was lower for men at all ages and for women age 56 and below when compared to the linear-quadratic model. The optimal BMI in the linear-quadratic model was 31.84 (95% CI, 30.34 to 33.34) for men and 23.04 (95% CI, 18.15 to 27.93) for women. With the categorical model, minimum mortality was associated with the overweight category for both genders.

### Mortality Estimates

Mortality estimates for a 50-year old individual across models are in Table 3. At the optimal BMI, the MFP model produced lower mortality estimates compared to the linear-quadratic model, but higher mortality estimates compared to the categorical model for men. For women, the MFP model produced lower mortality estimates compared to both the linear-quadratic and categorical models. Based on the MFP model, 5-year mortality for a 50-year old never smoker with BMI = 50 was 3.11 times greater than the minimum in the male sample and 3.54 times greater in the female sample. Mortality for an individual with BMI = 50 relative to the minimum was smaller in both the linear-quadratic and categorical models. For male never smokers, adjusted mortality was greater by a factor of 2.89 in the linear-quadratic model and a factor of 2.10 in the categorical model. For female never smokers, adjusted mortality was higher by a factor of 2.39 in the linear-quadratic model and a factor of 2.43 in the categorical model.

**Table 3:** Comparison of optimal BMI and 5-year mortality estimates across models

	MFP	Linear-Quadratic	Categorical
**Never Smokers**			

*Male*			

Optimal BMI	26.97 [26.41, 27.54]	31.84 [30.34, 33.34]	25-30

Mortality at Optimum	0.0176 [0.0158, 0.0196]	0.0188 [0.0168, 0.0210]	0.0169 [0.0152, 0.0188]

Mortality at BMI = 50	0.0548 [0.0428, 0.0699]	0.0544 [0.0383, 0.0766]	0.0355 [0.0251, 0.0499]

*Female*			

Optimal BMI	22.34 [20.10, 24.57]	23.04 [18.15, 27.93]	25-30

Mortality at Optimum	0.0100 [0.0088, 0.0115]	0.0128 [0.0115, 0.0141]	0.0120 [0.0107, 0.0134]

Mortality at BMI = 50	0.0355 [0.0274, 0.0460]	0.0306 [0.0377, 0.0248]	0.0291 [0.0233, 0.0363]

**Ever Smokers**			

*Male*			

Optimal BMI	26.97 [26.41, 27.54]	31.84 [30.34, 33.34]	25-30

Mortality at Optimum	0.0306 [0.0283, 0.0331]	0.0329 [0.0301, 0.0358]	0.0299 [0.0273, 0.0326]

Mortality at BMI = 50	0.0939 [0.0750, 0.1168]	0.0926 [0.0667, 0.1271]	0.0619 [0.0447, 0.0852]

*Female*			

Optimal BMI	22.34 [20.10, 24.57]	23.04 [18.15, 27.93]	25-30

Mortality at Optimum	0.0223 [0.0199, 0.0250]	0.0237 [0.0217, 0.0260]	0.0223 [0.0200, 0.0248]

Mortality at BMI = 50	0.0765 [0.0610, 0.0955]	0.0559 [0.0459, 0.0680]	0.0534 [0.0433, 0.0657]

95% confidence intervals are in brackets.

## Discussion

This paper outlined and applied a flexible method to modelling the nonlinear and asymmetric relationship between BMI and mortality. Using the MFP approach, we found that the BMI-mortality relation was J-shaped for women and U-shaped for men among individuals with a BMI of 18.5 and over. We also identified the nadir of the BMI-mortality curve to exist in the overweight range for the average U.S. male and the normal range for the average U.S female. However, differences in death probabilities around the nadir were small. The results in this paper with regard to the shape and nadir [2,10,25,34,35] of the BMI-mortality curve are consistent with prior findings. With regard to the nadir, most studies have found the nadir is in the normal BMI category, however, minimum mortality associated with the overweight category has been found in a number of other studies. For example, using NHANES I data, Durazo et al. (1997) [34] reported the BMI of minimum mortality to be 24.8 for white men and 24.3 for white women. However, for black men and women, the BMI of minimum mortality was 27.1 and 26.8, respectively. The downward slope at the low end of the BMI distribution for men stemmed from the fact that the normal BMI category consists of a mix of healthy lean and chronically ill, which confounded the relationship between mortality and obesity. This result was consistent with other studies [36-38] that report an inverse relation between low BMI individuals and mortality irrespective of the length of follow-up.

The MFP approach provides a robust alternative to other commonly used methods for addressing the nonlinear and asymmetric relationship between BMI and mortality by allowing the data itself to determine the functional form for BMI and other adjustment variables. The closed test algorithm used in this study determined the best fitting model from a predefined set of candidate models based on power transformations of BMI. Improvements in model fit were found relative to other commonly used models including the linear-quadratic BMI model, which imposes symmetry on the BMI-mortality curve. This in conjunction with low variation in mortality among those in the 21-30 BMI range and high mortality among those with BMI over 40 resulted in an overly flat curve in the center. Allowing the estimated curve to have a flexible shape made the MFP model more sensitive to variation in death rates in the data. Improvements in model fit were generally accompanied by smaller estimates of the BMI associated with minimum mortality and narrower confidence intervals. While the differences in optimal BMI were small for the representative 50-year old female, there was a large discrepancy in the male sample with the nadir extending into the class I obese range (BMI 30.0-34.9).

The other common approach to modelling the nonlinear functional form for BMI is a nonparametric approach incorporating categorical variables defined by WHO BMI classifications. Assessing the risk of a BMI category relative to the normal category is a convenient method to account for the nonlinear form, but assumes mortality is uniform across a BMI category, which is problematic when a category is heterogenous. In particular, the normal category consists of a mix of healthy and sick lean. Studies employing the categorical approach also typically take the mortality risk of obese individuals beyond a given threshold as constant. We addressed this difficulty by allowing the data from across the entire BMI distribution to predict mortality risk at extreme obesity levels, where fewer observations exist. Because our results showed that mortality increased exponentially for extremely obese individuals, categorization can drastically underestimate mortality at the right tail of the BMI distribution. The use of wide BMI categories are also inadequate for the purposes of prediction. Categorizing BMI using finer intervals can alleviate some of these difficulties, but the decision of which categories to add is generally an arbitrary choice. Moreover, the additional categories increase the variance of parameter estimates, particularly in high BMI categories where the sample size is small.

Our study also employed methods that differentiate the effect of BMI across gender and age. The identification of distinct FP terms across gender samples point to important differences in the BMI-mortality relationship. Further differences were identified within the female sample through interactions. The finding of significant interactions between age and BMI is not common in literature, but has been identified in a select number of studies [39,40]. The inclusion of age-BMI interactions resulted in optimal BMI estimates that varied with age and predicted the nadir to exist in the overweight range for women at age 71 and above.

This study has at least two notable limitations. The logistic regression model with 5-year mortality as the dependent outcome was chosen over the Cox survival model because the assumption of proportional hazards failed to hold. However, the disadvantage of the logistic regression model is that full information regarding the individual's exact time of death was not used. Second, while the primary goal of this study was to compare approaches to modelling the BMI-mortality relation using three important covariables, the complete case approach to addressing missing data and the omission of other potentially important explanatory variables may have introduced biases in parameter estimates.

## Conclusions

The MFP method identified improvements in model fit compared to other commonly employed models that estimate the BMI-mortality relationship, and is a robust method to determine the functional form for BMI. Using the MFP method, we found that the shape of the BMI-mortality curve was different across gender, but consistent with other previous studies. Specifically, the relation was U-shaped for men and J-shaped for women. We also identified important differences in shape and nadir of the BMI-mortality curve and mortality estimates compared to other commonly used models. Understanding the relation between obesity and BMI is important from a policy perspective, for addressing issues such as determining the efficacy of approaches designed to reduce obesity and in communicating with the public about the importance of obesity as a public health issue. Flexible methods, such as those employed in this study, are central in achieving reliability in measures used relevant analyses and are also potentially useful in estimating the relationship between the full spectrum of BMI values and other health outcomes or costs.

## Competing interests

The authors declare that they have no competing interests.

## Authors' contributions

ESW conceived of the study, participated in its design, performed statistical analyses and drafted the manuscript. BCMW, LPG, RAC and DEA participated in the design of the study and helped to draft the manuscript. DRF and SDS participated in the design of the study, helped to draft the manuscript and acquired funding for the project. All authors read and approved the final manuscript.

## Pre-publication history

The pre-publication history for this paper can be accessed here:

http://www.biomedcentral.com/1471-2288/11/175/prepub
